# Supplementary material for: Child Domestic Work, Violence, and Health Outcomes: A Rapid Systematic Review
Source: Int J Environ Res Public Health. 2021 Dec 31;19(1):427. doi: 10.3390/ijerph19010427 (PMC8744913; doi:10.3390/ijerph19010427)
Supplement: Supplementary file 1 [file ijerph-19-00427-s001.zip › ijerph-1494951 -supplementary1.pdf]

Supplementary File 1. Search strategy methodology and results

Contents

1 Search methodology 1
2 Search results 2
3 Search strategies 2
3.1 OvidSP Medline 2
3.2 OvidSP Embase 7
3.3 OvidSP Medline In-Process & Other Non-Indexed Citations and Daily updates 12
3.4 OvidSP Global Health 17
3.5 OvidSP EconLit 22
3.6 Web of Science Core Collection 23
3.7 IBSS 24
3.8 ILO Labourdoc (grey) 26

1 Search methodology

Seven databases were searched on 05 June 2019. The search strategies looked for information on child, migrant or foreign domestic workers, with studies set in a named high-income country removed. The search strategy was compiled in the OvidSP Medline database by Jane Falconer, and refined with the project team until the results retrieved reflected the scope of the project. The final Medline search was amended to run across the other databases.

As the vocabulary used for this search is very common across a variety of subject areas, and there are no subject heading terms which adequately cover child domestic workers, migrant domestic workers or foreign domestic workers, a mixture of proximity searching and focused subject headings were used, where available. The list of high income countries was derived from the World Bank country classifications from July 2019.

The databases searched were:

1. OvidSP Medline and In-Process & Other Non-Indexed Citations and Daily, 1946 to June 04, 2019.
2. OvidSP Embase+Embase Classic, 1947 to 2019 June 04.
3. OvidSP Global Health, 1910 to 2019 week 21
4. OvidSP EconLit, 1886 to May 23, 2019
5. Web of Science Core Collection, which included:
    a. Science Citation Index Expanded, 1970 – 2019/06/04
    b. Social Sciences Citation Index, 1970 – 2019/06/04
    c. Arts & Humanities Citation Index, 1975 – 2019/06/04
    d. Conference Proceedings Citation Index- Science, 1990 – 2019/06/04
    e. Conference Proceedings Citation Index- Social Science & Humanities, 1990 – 2019/06/04
    f. Emerging Sources Citation Index, 2015 – 2019/06/04
6. ProQuest International Bibliography of the Social Sciences (IBSS), complete database as of 05/06/2019.

Complete search strategies are provided below.

2 Search results

Search results were uploaded to EndNote X9 and deduplicated. using a technique developed at the University of Leeds.[1] Number of results pre- and post- deduplication are listed in the table below.

Database name	EndNote import order	Number of references before deduplication	Number of references after deduplication
Medline	1	1,166	1,164
Embase	2	2,773	2,126
Medline in process[2]	3	116	82
Global Health	4	1,875	1,593
EconLit	5	483	454
Web of Science	6	2,224	1,523
IBSS	7	1,400	472
**Total**		10,036	7,414

3 Search strategies

3.1 OvidSP Medline

Database name	Medline
Database platform	OvidSP
Dates of database coverage	1946 to June 04, 2019
Date searched	05/06/2019
Searched by	JF
Number of hits	1166

1 child/ (1618012)
2 adolescent/ (1936326)
3 young adult/ (746037)
4 minors/ (2504)

[1] Deduplication method is published at http://medhealth.leeds.ac.uk/download/2518/auhe_duplicate_checking_guide.
[2] Medline results are split into Medline and Medline In-Process & Other Non-Indexed Citations and Daily updates to allow automatic deduplication to retain potential duplicates from Embase.

5 Child, Orphaned/ (659)
6 child, foster/ (71)
7 "transients and migrants"/ (10785)
8 or/1-7 [MeSH people] (3033207)
9 work/ (19653)
10 employment/ (44212)
11 social support/ (66790)
12 enslaved persons/ (47)
13 or/9-12 [MeSH work] (128283)
14 housing/ (16692)
15 child labor/ (8)
16 housekeeping/ (1604)
17 *child/ (3410)
18 *adolescent/ (5534)
19 *young adult/ (93)
20 *minors/ (1426)
21 *Child, Orphaned/ (507)
22 *child, foster/ (55)
23 *"transients and migrants"/ (7645)
24 or/17-23 [focused MeSH people] (17737)
25 *work/ (15839)
26 *employment/ (24277)
27 *social support/ (24935)
28 *enslaved persons/ (41)
29 or/25-28 [focused MeSH work] (64525)
30 *housing/ (8269)
31 *child labor/ (4)
32 *housekeeping/ (957)
33 ((child* or boy? or girl? or schoolage? or schoolchild* or prepubescen* or pubescen* or puberty or adolescen* or juvenil* or underage* or teen or teens or teenage* or preteen* or pre-teen* or youth? or early adult* or emerging adult* or young adult* or young person or young people or minor? or offspring or sibling* or son or sons or daughter? or orphan* or migrant* or foreign* or transient) adj3 (labo?r* or physical* demand* or workday? or work or working or worker? or service? or exploit* or slavery or slave? or servitude or livelihood? or employ* or chores or cleaning or cooking or child-care or childcare)).ti,ab. (43877)
34 ((child* or boy? or girl? or schoolage? or schoolchild* or prepubescen* or pubescen* or puberty or adolescen* or juvenil* or underage* or teen or teens or teenage* or preteen* or pre-teen* or youth? or early adult* or emerging adult* or young adult* or young person or young people or minor? or offspring or sibling* or son or sons or daughter? or orphan* or migrant* or foreign* or transient) adj3 (domestic or household? or home or house or kin relations* or kinship or fostering or relocation or non-parental residence)).ti,ab. (14697)
35 ((labo?r* or physical* demand* or workday? or work or working or worker? or service? or exploit* or slavery or slave? or servitude or livelihood? or employ* or chores or cleaning or cooking or child-care or childcare) adj3 (domestic or household? or home or house or kin relations* or kinship or fostering or relocation or non-parental residence)).ti,ab. (18863)
36 (child* or boy? or girl? or schoolage? or schoolchild* or prepubescen* or pubescen* or puberty or adolescen* or juvenil* or underage* or teen or teens or teenage* or preteen* or pre-teen* or youth? or early adult* or emerging adult* or young adult* or young person or young people or minor? or offspring or sibling* or son or sons or daughter? or orphan* or migrant* or foreign* or transient).ti,ab. (2374217)

37 (labo?r* or physical* demand* or workday? or work or working or worker? or service? or exploit* or slavery or slave? or servitude or livelihood? or employ* or chores or cleaning or cooking or child-care or childcare).ti,ab. (2722543)

38 (domestic or household? or home or house or kin relations* or kinship or fostering or relocation or non-parental residence).ti,ab. (397464)

39 (housework or maid? or housemaid? or house help or domestic help).ti,ab. (1873)

40 8 and 13 and 14 (290)

41 14 and 15 (0)

42 8 and 16 (304)

43 30 and 33 (86)

44 29 and 34 (336)

45 24 and 35 (231)

46 32 and 36 (149)

47 31 and 39 (0)

48 29 and 30 and 36 (59)

49 24 and 30 and 37 (63)

50 24 and 29 and 38 (203)

51 ((child* or boy? or girl? or schoolage? or schoolchild* or prepubescen* or pubescen* or puberty or adolescen* or juvenil* or underage* or teen or teens or teenage* or preteen* or pre-teen* or youth? or early adult* or emerging adult* or young adult* or young person or young people or minor? or offspring or sibling* or son or sons or daughter? or orphan* or migrant* or foreign* or transient) adj3 (labo?r* or physical* demand* or workday? or work or working or worker? or service? or exploit* or slavery or slave? or servitude or livelihood? or employ* or chores or cleaning or cooking or child-care or childcare) adj3 (domestic or household? or home or house or kin relations* or kinship or fostering or relocation or non-parental residence)).ti,ab. (970)

52 ((child* or boy? or girl? or schoolage? or schoolchild* or prepubescen* or pubescen* or puberty or adolescen* or juvenil* or underage* or teen or teens or teenage* or preteen* or pre-teen* or youth? or early adult* or emerging adult* or young adult* or young person or young people or minor? or offspring or sibling* or son or sons or daughter? or orphan* or migrant* or foreign* or transient) adj3 (housework or maid? or housemaid? or house help or domestic help)).ti,ab. (115)

53 or/40-52 (2325)

54 exp Australia/ (135086)

55 australia.ti,ab. (85237)

56 french polynesia.ti,ab. (892)

57 micronesia/ or guam/ (1746)

58 (guam or northern mariana islands).ti,ab. (1199)

59 New Caledonia/ (828)

60 new caledonia.ti,ab. (1365)

61 New Zealand/ (37277)

62 new zealand.ti,ab. (49537)

63 exp Japan/ (127597)

64 japan.ti,ab. (108382)

65 korea/ or "republic of korea"/ (40861)

66 ((korea or south korea or (republic adj2 korea)) not (north korea or (democratic adj2 korea))).ti,ab. (39951)

67 Palau/ (190)

68 bel (339)

69 Andorra/ (26)

70 andorra.ti,ab. (54)

71 Cyprus/ (1222)

72 cyprus.ti,ab. (1696)

73 Liechtenstein/ (39)
74 liechtenstein.ti,ab. (284)
75 Monaco/ (79)
76 monaco.ti,ab. (332)
77 San Marino/ (73)
78 san marino.ti,ab. (89)
79 Austria/ (18584)
80 austria.ti,ab. (11280)
81 Belgium/ (17776)
82 belgium.ti,ab. (14374)
83 Czech Republic/ (7096)
84 czech republic.ti,ab. (7308)
85 Denmark/ (46535)
86 (denmark or faeroe islands).ti,ab. (25224)
87 Sweden/ (70441)
88 sweden.ti,ab. (42545)
89 exp Norway/ (37094)
90 norway.ti,ab. (28310)
91 Scandinavia/ or exp "Scandinavian and Nordic Countries"/ (191757)
92 scandinavia.ti,ab. (2588)
93 exp Baltic States/ (6594)
94 estonia.ti,ab. (2353)
95 latvia.ti,ab. (1199)
96 lithuania.ti,ab. (2353)
97 Finland/ (33784)
98 finland.ti,ab. (22976)
99 exp France/ (96382)
100 france.ti,ab. (57161)
101 exp Germany/ (112408)
102 germany.ti,ab. (72989)
103 Gibraltar/ (96)
104 gibraltar.ti,ab. (342)
105 Greece/ (16015)
106 greece.ti,ab. (13525)
107 Greenland/ (2318)
108 greenland.ti,ab. (3404)
109 Hungary/ (17979)
110 hungary.ti,ab. (9806)
111 Iceland/ (4201)
112 iceland.ti,ab. (4402)
113 Ireland/ (16874)
114 (ireland or eire).ti,ab. (19377)
115 exp Italy/ (89956)
116 italy.ti,ab. (57566)
117 Luxembourg/ (682)
118 luxembourg.ti,ab. (939)
119 Netherlands/ (63960)
120 (netherlands or holland).ti,ab. (46302)
121 Poland/ (46155)

122 poland.ti,ab. (22110)
123 Portugal/ (11878)
124 portugal.ti,ab. (10660)
125 Slovakia/ (2719)
126 (slovakia or slovak republic).ti,ab. (3797)
127 Slovenia/ (2465)
128 slovenia.ti,ab. (3055)
129 Spain/ (71767)
130 (spain or balearic islands or canary islands).ti,ab. (51719)
131 Switzerland/ (34230)
132 switzerland.ti,ab. (21805)
133 exp Great Britain/ (353098)
134 (great britain or GBR or united kingdom or UK or northern ireland or scotland or channel islands or isle of man or (wales not new south wales) or (england not new england)).ti,ab. (187988)
135 "Antigua and Barbuda"/ (107)
136 (Antigua or Barbuda).ti,ab. (223)
137 Argentina/ (14675)
138 argentina.ti,ab. (15156)
139 West Indies/ (3441)
140 Netherlands Antilles/ (286)
141 aruba.ti,ab. (171)
142 Bahamas/ (442)
143 bahamas.ti,ab. (666)
144 Barbados/ (603)
145 barbados.ti,ab. (936)
146 british virgin islands/ (5)
147 british virgin islands.ti,ab. (52)
148 cayman islands.ti,ab. (77)
149 Chile/ (12790)
150 Chile.ti,ab. (12123)
151 curacao.ti,ab. (382)
152 Puerto Rico/ (6082)
153 puerto rico.ti,ab. (5997)
154 (st martin or sint maarten).ti,ab. (83)
155 "Trinidad and Tobago"/ (1707)
156 (trinidad adj2 tobago).ti,ab. (777)
157 (turks adj2 caicos islands).ti,ab. (19)
158 "Virgin Islands of the United States"/ (272)
159 virgin islands.ti,ab. (527)
160 Uruguay/ (1763)
161 Uruguay.ti,ab. (2563)
162 "Saint Kitts and Nevis"/ (63)
163 ((st kitts or saint kitts) adj2 nevis).ti,ab. (60)
164 Panama/ (2234)
165 panama.ti,ab. (3764)
166 Seychelles/ (349)
167 Seychelles.ti,ab. (660)
168 Bahrain/ (567)
169 bahrain.ti,ab. (808)

170 Israel/ (27884)
171 Israel.ti,ab. (21300)
172 Kuwait/ (2926)
173 kuwait.ti,ab. (3211)
174 Malta/ (713)
175 Malta.ti,ab. (1189)
176 Oman/ (1346)
177 oman.ti,ab. (2410)
178 Qatar/ (1003)
179 (qatar or quatar or katar).ti,ab. (1545)
180 Saudi Arabia/ (11892)
181 saudi arabia.ti,ab. (14108)
182 United Arab Emirates/ (1837)
183 (united arab emirates or trucial states or abu dhabi or ajman or dubai or fujairah or ras al-khaimah or sharjah or umm al-qaiwain).ti,ab. (2477)
184 Bermuda/ (176)
185 bermuda.ti,ab. (865)
186 exp Canada/ (149863)
187 canada.ti,ab. (77148)
188 exp United States/ (1297837)
189 (united states or (united states adj2 america) or USA).ti,ab. (310373)
190 or/54-189 (3496167)
191 53 not 190 (1282)
192 limit 191 to medline (1166)

3.2 OvidSP Embase

Database name	Embase+Embase Classic
Database platform	OvidSP
Dates of database coverage	1947 to 2019 June 04
Date searched	05/06/2019
Searched by	JF
Number of hits	2773

1 child/ (1853649)
2 adopted child/ (555)
3 foster child/ (69)
4 orphaned child/ (605)
5 adolescent/ (1573447)
6 juvenile/ (45107)
7 young adult/ (292889)
8 boy/ (45218)
9 girl/ (58997)
10 "minor (person)"/ (561)

11 migrant/ (6279)
12 or/1-11 (2960796)
13 work/ (36446)
14 exp employment/ (92068)
15 working poor/ (41)
16 cleaning/ (11956)
17 child care/ (36899)
18 infant care/ (808)
19 social support/ (84489)
20 slave/ (136)
21 or/13-20 (253610)
22 housing/ (25043)
23 household/ (36187)
24 foster care/ (4748)
25 or/22-24 (64982)
26 child labor/ (151)
27 migrant worker/ (1512)
28 or/26-27 (1662)
29 housekeeping/ (609)
30 *child/ (130222)
31 *adopted child/ (184)
32 *foster child/ (19)
33 *orphaned child/ (307)
34 *adolescent/ (30826)
35 *juvenile/ (14603)
36 *young adult/ (2761)
37 *boy/ (3006)
38 *girl/ (5784)
39 *"minor (person)"/ (167)
40 *migrant/ (2285)
41 or/30-40 (176467)
42 *work/ (12962)
43 exp *employment/ (25784)
44 *working poor/ (17)
45 *cleaning/ (2405)
46 *child care/ (14825)
47 *infant care/ (282)
48 *social support/ (20774)
49 *slave/ (22)
50 or/42-49 (76352)
51 *household/ (4241)
52 *foster care/ (2358)
53 *housing/ (8479)
54 or/51-53 (15036)
55 *child labor/ (112)
56 *migrant worker/ (699)
57 or/55-56 (811)
58 *housekeeping/ (45)

59 ((child* or boy? or girl? or schoolage? or schoolchild* or prepubescen* or pubescen* or puberty or adolescen* or juvenil* or underage* or teen or teens or teenage* or preteen* or pre-teen* or youth? or early adult* or emerging adult* or young adult* or young person or young people or minor? or offspring or sibling* or son or sons or daughter? or orphan* or migrant* or foreign* or transient) adj3 (labo?r* or physical* demand* or workday? or work or working or worker? or service? or exploit* or slavery or slave? or servitude or livelihood? or employ* or chores or cleaning or cooking or child-care or childcare)).ti,ab. (56287)
60 ((child* or boy? or girl? or schoolage? or schoolchild* or prepubescen* or pubescen* or puberty or adolescen* or juvenil* or underage* or teen or teens or teenage* or preteen* or pre-teen* or youth? or early adult* or emerging adult* or young adult* or young person or young people or minor? or offspring or sibling* or son or sons or daughter? or orphan* or migrant* or foreign* or transient) adj3 (domestic or household? or home or house or kin relations* or kinship or fostering or relocation or non-parental residence)).ti,ab. (18673)
61 ((labo?r* or physical* demand* or workday? or work or working or worker? or service? or exploit* or slavery or slave? or servitude or livelihood? or employ* or chores or cleaning or cooking or child-care or childcare) adj3 (domestic or household? or home or house or kin relations* or kinship or fostering or relocation or non-parental residence)).ti,ab. (24638)
62 (child* or boy? or girl? or schoolage? or schoolchild* or prepubescen* or pubescen* or puberty or adolescen* or juvenil* or underage* or teen or teens or teenage* or preteen* or pre-teen* or youth? or early adult* or emerging adult* or young adult* or young person or young people or minor? or offspring or sibling* or son or sons or daughter? or orphan* or migrant* or foreign* or transient).ti,ab. (3272271)
63 (labo?r* or physical* demand* or workday? or work or working or worker? or service? or exploit* or slavery or slave? or servitude or livelihood? or employ* or chores or cleaning or cooking or child-care or child-care).ti,ab. (3639488)
64 (domestic or household? or home or house or kin relations* or kinship or fostering or relocation or non-parental residence).ti,ab. (548894)
65 (housework or maid? or housemaid? or house help or domestic help).ti,ab. (2455)
66 12 and 21 and 25 (2100)
67 25 and 28 (63)
68 12 and 29 (75)
69 54 and 59 (436)
70 50 and 60 (674)
71 41 and 61 (256)
72 58 and 62 (7)
73 57 and 65 (5)
74 50 and 54 and 62 (219)
75 41 and 54 and 63 (106)
76 41 and 50 and 64 (92)
77 ((child* or boy? or girl? or schoolage? or schoolchild* or prepubescen* or pubescen* or puberty or adolescen* or juvenil* or underage* or teen or teens or teenage* or preteen* or pre-teen* or youth? or early adult* or emerging adult* or young adult* or young person or young people or minor? or offspring or sibling* or son or sons or daughter? or orphan* or migrant* or foreign* or transient) adj3 (labo?r* or physical* demand* or workday? or work or working or worker? or service? or exploit* or slavery or slave? or servitude or livelihood? or employ* or chores or cleaning or cooking or child-care or childcare) adj3 (domestic or household? or home or house or kin relations* or kinship or fostering or relocation or non-parental residence)).ti,ab. (1086)
78 ((child* or boy? or girl? or schoolage? or schoolchild* or prepubescen* or pubescen* or puberty or adolescen* or juvenil* or underage* or teen or teens or teenage* or preteen* or pre-teen* or youth? or early adult* or emerging adult* or young adult* or young person or young people or minor? or offspring or sibling* or son or sons or daughter? or orphan* or migrant* or foreign* or transient) adj3 (housework or maid? or housemaid? or house help or domestic help)).ti,ab. (119)
79 or/66-78 (4482)

80 Seychelles/ (407)
81 Seychelles.ti,ab. (686)
82 exp "Australia and New Zealand"/ (219879)
83 australia.ti,ab. (113707)
84 new zealand.ti,ab. (65821)
85 french polynesia/ (459)
86 french polynesia.ti,ab. (994)
87 Guam/ (953)
88 (guam or northern mariana islands).ti,ab. (1461)
89 New Caledonia/ (836)
90 new caledonia.ti,ab. (1444)
91 Japan/ (172422)
92 japan.ti,ab. (186585)
93 korea/ or south korea/ (58007)
94 ((korea or south korea or (republic adj2 korea)) not (north korea or (democratic adj2 korea))).ti,ab. (55890)
95 Palau/ (270)
96 (palau or paulau).ti,ab. (382)
97 Andorra/ (46)
98 andorra.ti,ab. (66)
99 Cyprus/ (2195)
100 cyprus.ti,ab. (2354)
101 Liechtenstein/ (121)
102 liechtenstein.ti,ab. (333)
103 monaco/ (672)
104 monaco.ti,ab. (959)
105 San Marino/ (116)
106 san marino.ti,ab. (123)
107 Austria/ (23295)
108 austria.ti,ab. (17907)
109 exp Belgium/ (25323)
110 belgium.ti,ab. (21638)
111 Czech Republic/ (13010)
112 czech republic.ti,ab. (13005)
113 exp Scandinavia/ (208585)
114 (denmark or faeroe islands).ti,ab. (34293)
115 sweden.ti,ab. (57409)
116 norway.ti,ab. (36047)
117 scandinavia.ti,ab. (3372)
118 finland.ti,ab. (29390)
119 iceland.ti,ab. (5339)
120 exp Baltic States/ (9523)
121 estonia.ti,ab. (3302)
122 latvia.ti,ab. (1953)
123 lithuania.ti,ab. (3300)
124 baltic states.ti,ab. (229)
125 exp France/ (126720)
126 france.ti,ab. (90319)
127 exp Germany/ (191578)
128 germany.ti,ab. (116980)

129 Gibraltar/ (148)
130 gibraltar.ti,ab. (339)
131 Greece/ (26901)
132 greece.ti,ab. (19351)
133 Greenland/ (3022)
134 greenland.ti,ab. (3695)
135 Hungary/ (22304)
136 hungary.ti,ab. (16720)
137 Ireland/ (34093)
138 (ireland or eire).ti,ab. (163861)
139 exp Italy/ (113503)
140 italy.ti,ab. (81214)
141 Luxembourg/ (1290)
142 luxembourg.ti,ab. (1241)
143 Netherlands/ (80374)
144 (netherlands or holland).ti,ab. (68229)
145 Poland/ (53334)
146 poland.ti,ab. (31770)
147 exp Portugal/ (18484)
148 portugal.ti,ab. (16098)
149 Slovakia/ (5382)
150 (slovakia or slovak republic).ti,ab. (5887)
151 Slovenia/ (4821)
152 slovenia.ti,ab. (4903)
153 exp Spain/ (90753)
154 (spain or balearic islands or canary islands).ti,ab. (70998)
155 Switzerland/ (41369)
156 switzerland.ti,ab. (45138)
157 exp United Kingdom/ (433402)
158 (great britain or GBR or united kingdom or UK or northern ireland or scotland or channel islands or isle of man or (wales not new south wales) or (england not new england)).ti,ab. (334506)
159 "Antigua and Barbuda"/ (164)
160 (Antigua or Barbuda).ti,ab. (239)
161 Argentina/ (21323)
162 argentina.ti,ab. (20566)
163 aruba/ (92)
164 aruba.ti,ab. (234)
165 Bahamas/ (707)
166 bahamas.ti,ab. (854)
167 Barbados/ (791)
168 barbados.ti,ab. (1109)
169 "virgin islands (british)"/ (43)
170 british virgin islands.ti,ab. (55)
171 cayman islands/ (59)
172 cayman islands.ti,ab. (92)
173 Chile/ (16329)
174 chile.ti,ab. (14664)
175 curacao/ (149)
176 curacao.ti,ab. (617)

177 Puerto Rico/ (7476)
178 puerto rico.ti,ab. (7256)
179 exp saint martin/ (97)
180 (st martin or sint maarten).ti,ab. (101)
181 "Trinidad and Tobago"/ (1583)
182 (trinidad adj2 tobago).ti,ab. (940)
183 "turks and caicos islands"/ (20)
184 (turks adj2 caicos islands).ti,ab. (28)
185 "Virgin Islands (U.S.)"/ (258)
186 virgin islands.ti,ab. (578)
187 Uruguay/ (2990)
188 uruguay.ti,ab. (3278)
189 "Saint Kitts and Nevis"/ (72)
190 ((st kitts or saint kitts) adj2 nevis).ti,ab. (74)
191 Panama/ (3164)
192 panama.ti,ab. (4107)
193 Seychelles/ (407)
194 seychelles.ti,ab. (686)
195 Bahrain/ (1327)
196 bahrain.ti,ab. (1400)
197 Israel/ (31483)
198 israel.ti,ab. (27130)
199 Kuwait/ (4686)
200 kuwait.ti,ab. (4697)
201 Malta/ (1629)
202 malta.ti,ab. (1917)
203 Oman/ (2496)
204 oman.ti,ab. (2635)
205 Qatar/ (2468)
206 (qatar or quatar or katar).ti,ab. (2570)
207 Saudi Arabia/ (19396)
208 saudi arabia.ti,ab. (17678)
209 exp United Arab Emirates/ (3291)
210 (united arab emirates or trucial states or abu dhabi or ajman or dubai or fujairah or ras al-khaimah or sharjah or umm al-qaiwain).ti,ab. (3381)
211 Bermuda/ (406)
212 bermuda.ti,ab. (1069)
213 exp Canada/ (180247)
214 exp United States/ (1256605)
215 (united states or (united states adj2 america) or USA).ti,ab. (469487)
216 or/80-215 (4103198)
217 79 not 216 (2773)

3.3 OvidSP Medline In-Process & Other Non-Indexed Citations and Daily updates

Database name	Ovid MEDLINE(R) and In-Process & Other Non-Indexed Citations and Daily
Database platform	OvidSP

Dates of database coverage	1946 to June 04, 2019
Date searched	05/06/2019
Searched by	JF
Number of hits	116

1 child/ (1618012)
2 adolescent/ (1936326)
3 young adult/ (746037)
4 minors/ (2504)
5 Child, Orphaned/ (659)
6 child, foster/ (71)
7 "transients and migrants"/ (10785)
8 or/1-7 [MeSH people] (3033207)
9 work/ (19653)
10 employment/ (44212)
11 social support/ (66790)
12 enslaved persons/ (47)
13 or/9-12 [MeSH work] (128283)
14 housing/ (16692)
15 child labor/ (8)
16 housekeeping/ (1604)
17 *child/ (3410)
18 *adolescent/ (5534)
19 *young adult/ (93)
20 *minors/ (1426)
21 *Child, Orphaned/ (507)
22 *child, foster/ (55)
23 *"transients and migrants"/ (7645)
24 or/17-23 [focused MeSH people] (17737)
25 *work/ (15839)
26 *employment/ (24277)
27 *social support/ (24935)
28 *enslaved persons/ (41)
29 or/25-28 [focused MeSH work] (64525)
30 *housing/ (8269)
31 *child labor/ (4)
32 *housekeeping/ (957)
33 ((child* or boy? or girl? or schoolage? or schoolchild* or prepubescen* or pubescen* or puberty or adolescen* or juvenil* or underage* or teen or teens or teenage* or preteen* or pre-teen* or youth? or early adult* or emerging adult* or young adult* or young person or young people or minor? or offspring or sibling* or son or sons or daughter? or orphan* or migrant* or foreign* or transient) adj3 (labo?r* or physical* demand* or workday? or work or working or worker? or service? or exploit* or slavery or slave? or servitude or livelihood? or employ* or chores or cleaning or cooking or child-care or childcare)).ti,ab. (43877)
34 ((child* or boy? or girl? or schoolage? or schoolchild* or prepubescen* or pubescen* or puberty or adolescen* or juvenil* or underage* or teen or teens or teenage* or preteen* or pre-teen* or youth? or early adult* or

emerging adult* or young adult* or young person or young people or minor? or offspring or sibling* or son or sons or daughter? or orphan* or migrant* or foreign* or transient) adj3 (domestic or household? or home or house or kin relations* or kinship or fostering or relocation or non-parental residence)).ti,ab. (14697)

35 ((labo?r* or physical* demand* or workday? or work or working or worker? or service? or exploit* or slavery or slave? or servitude or livelihood? or employ* or chores or cleaning or cooking or child-care or childcare) adj3 (domestic or household? or home or house or kin relations* or kinship or fostering or relocation or non-parental residence)).ti,ab. (18863)

36 (child* or boy? or girl? or schoolage? or schoolchild* or prepubescen* or pubescen* or puberty or adolescen* or juvenil* or underage* or teen or teens or teenage* or preteen* or pre-teen* or youth? or early adult* or emerging adult* or young adult* or young person or young people or minor? or offspring or sibling* or son or sons or daughter? or orphan* or migrant* or foreign* or transient).ti,ab. (2374217)

37 (labo?r* or physical* demand* or workday? or work or working or worker? or service? or exploit* or slavery or slave? or servitude or livelihood? or employ* or chores or cleaning or cooking or child-care or child-care).ti,ab. (2722543)

38 (domestic or household? or home or house or kin relations* or kinship or fostering or relocation or non-parental residence).ti,ab. (397464)

39 (housework or maid? or housemaid? or house help or domestic help).ti,ab. (1873)

40 8 and 13 and 14 (290)

41 14 and 15 (0)

42 8 and 16 (304)

43 30 and 33 (86)

44 29 and 34 (336)

45 24 and 35 (231)

46 32 and 36 (149)

47 31 and 39 (0)

48 29 and 30 and 36 (59)

49 24 and 30 and 37 (63)

50 24 and 29 and 38 (203)

51 ((child* or boy? or girl? or schoolage? or schoolchild* or prepubescen* or pubescen* or puberty or adolescen* or juvenil* or underage* or teen or teens or teenage* or preteen* or pre-teen* or youth? or early adult* or emerging adult* or young adult* or young person or young people or minor? or offspring or sibling* or son or sons or daughter? or orphan* or migrant* or foreign* or transient) adj3 (labo?r* or physical* demand* or workday? or work or working or worker? or service? or exploit* or slavery or slave? or servitude or livelihood? or employ* or chores or cleaning or cooking or child-care or childcare) adj3 (domestic or household? or home or house or kin relations* or kinship or fostering or relocation or non-parental residence)).ti,ab. (970)

52 ((child* or boy? or girl? or schoolage? or schoolchild* or prepubescen* or pubescen* or puberty or adolescen* or juvenil* or underage* or teen or teens or teenage* or preteen* or pre-teen* or youth? or early adult* or emerging adult* or young adult* or young person or young people or minor? or offspring or sibling* or son or sons or daughter? or orphan* or migrant* or foreign* or transient) adj3 (housework or maid? or housemaid? or house help or domestic help)).ti,ab. (115)

53 or/40-52 (2325)

54 exp Australia/ (135086)

55 australia.ti,ab. (85237)

56 french polynesia.ti,ab. (892)

57 micronesia/ or guam/ (1746)

58 (guam or northern mariana islands).ti,ab. (1199)

59 New Caledonia/ (828)

60 new caledonia.ti,ab. (1365)

61 New Zealand/ (37277)

62 new zealand.ti,ab. (49537)
63 exp Japan/ (127597)
64 japan.ti,ab. (108382)
65 korea/ or "republic of korea"/ (40861)
66 ((korea or south korea or (republic adj2 korea)) not (north korea or (democratic adj2 korea))).ti,ab. (39951)
67 Palau/ (190)
68 bel (339)
69 Andorra/ (26)
70 andorra.ti,ab. (54)
71 Cyprus/ (1222)
72 cyprus.ti,ab. (1696)
73 Liechtenstein/ (39)
74 liechtenstein.ti,ab. (284)
75 Monaco/ (79)
76 monaco.ti,ab. (332)
77 San Marino/ (73)
78 san marino.ti,ab. (89)
79 Austria/ (18584)
80 austria.ti,ab. (11280)
81 Belgium/ (17776)
82 belgium.ti,ab. (14374)
83 Czech Republic/ (7096)
84 czech republic.ti,ab. (7308)
85 Denmark/ (46535)
86 (denmark or faeroe islands).ti,ab. (25224)
87 Sweden/ (70441)
88 sweden.ti,ab. (42545)
89 exp Norway/ (37094)
90 norway.ti,ab. (28310)
91 Scandinavia/ or exp "Scandinavian and Nordic Countries"/ (191757)
92 scandinavia.ti,ab. (2588)
93 exp Baltic States/ (6594)
94 estonia.ti,ab. (2353)
95 latvia.ti,ab. (1199)
96 lithuania.ti,ab. (2353)
97 Finland/ (33784)
98 finland.ti,ab. (22976)
99 exp France/ (96382)
100 france.ti,ab. (57161)
101 exp Germany/ (112408)
102 germany.ti,ab. (72989)
103 Gibraltar/ (96)
104 gibraltar.ti,ab. (342)
105 Greece/ (16015)
106 greece.ti,ab. (13525)
107 Greenland/ (2318)
108 greenland.ti,ab. (3404)
109 Hungary/ (17979)
110 hungary.ti,ab. (9806)

111 Iceland/ (4201)
112 iceland.ti,ab. (4402)
113 Ireland/ (16874)
114 (ireland or eire).ti,ab. (19377)
115 exp Italy/ (89956)
116 italy.ti,ab. (57566)
117 Luxembourg/ (682)
118 luxembourg.ti,ab. (939)
119 Netherlands/ (63960)
120 (netherlands or holland).ti,ab. (46302)
121 Poland/ (46155)
122 poland.ti,ab. (22110)
123 Portugal/ (11878)
124 portugal.ti,ab. (10660)
125 Slovakia/ (2719)
126 (slovakia or slovak republic).ti,ab. (3797)
127 Slovenia/ (2465)
128 slovenia.ti,ab. (3055)
129 Spain/ (71767)
130 (spain or balearic islands or canary islands).ti,ab. (51719)
131 Switzerland/ (34230)
132 switzerland.ti,ab. (21805)
133 exp Great Britain/ (353098)
134 (great britain or GBR or united kingdom or UK or northern ireland or scotland or channel islands or isle of man or (wales not new south wales) or (england not new england)).ti,ab. (187988)
135 "Antigua and Barbuda"/ (107)
136 (Antigua or Barbuda).ti,ab. (223)
137 Argentina/ (14675)
138 argentina.ti,ab. (15156)
139 West Indies/ (3441)
140 Netherlands Antilles/ (286)
141 aruba.ti,ab. (171)
142 Bahamas/ (442)
143 bahamas.ti,ab. (666)
144 Barbados/ (603)
145 barbados.ti,ab. (936)
146 british virgin islands/ (5)
147 british virgin islands.ti,ab. (52)
148 cayman islands.ti,ab. (77)
149 Chile/ (12790)
150 Chile.ti,ab. (12123)
151 curacao.ti,ab. (382)
152 Puerto Rico/ (6082)
153 puerto rico.ti,ab. (5997)
154 (st martin or sint maarten).ti,ab. (83)
155 "Trinidad and Tobago"/ (1707)
156 (trinidad adj2 tobago).ti,ab. (777)
157 (turks adj2 caicos islands).ti,ab. (19)
158 "Virgin Islands of the United States"/ (272)

159 virgin islands.ti,ab. (527)
160 Uruguay/ (1763)
161 Uruguay.ti,ab. (2563)
162 "Saint Kitts and Nevis"/ (63)
163 ((st kitts or saint kitts) adj2 nevis).ti,ab. (60)
164 Panama/ (2234)
165 panama.ti,ab. (3764)
166 Seychelles/ (349)
167 Seychelles.ti,ab. (660)
168 Bahrain/ (567)
169 bahrain.ti,ab. (808)
170 Israel/ (27884)
171 Israel.ti,ab. (21300)
172 Kuwait/ (2926)
173 kuwait.ti,ab. (3211)
174 Malta/ (713)
175 Malta.ti,ab. (1189)
176 Oman/ (1346)
177 oman.ti,ab. (2410)
178 Qatar/ (1003)
179 (qatar or quatar or katar).ti,ab. (1545)
180 Saudi Arabia/ (11892)
181 saudi arabia.ti,ab. (14108)
182 United Arab Emirates/ (1837)
183 (united arab emirates or trucial states or abu dhabi or ajman or dubai or fujairah or ras al-khaimah or sharjah or umm al-qaiwain).ti,ab. (2477)
184 Bermuda/ (176)
185 bermuda.ti,ab. (865)
186 exp Canada/ (149863)
187 canada.ti,ab. (77148)
188 exp United States/ (1297837)
189 (united states or (united states adj2 america) or USA).ti,ab. (310373)
190 or/54-189 (3496167)
191 53 not 190 (1282)
192 limit 191 to medline (1166)
193 191 not 192 (116)

3.4 OvidSP Global Health

Database name	Global Health
Database platform	OvidSP
Dates of database coverage	1910 to 2019 week 21
Date searched	05/06/2019
Searched by	JF

Number of hits	1875

1 children/ (336329)
2 adolescents/ (57651)
3 young adults/ (11383)
4 exp youth/ (14955)
5 boys/ (22903)
6 girls/ (28031)
7 adopted children/ (137)
8 foster children/ (108)
9 orphans/ (772)
10 migrants/ (3426)
11 or/1-10 (352316)
12 work/ (2074)
13 workers/ (40809)
14 employment/ (3643)
15 part time employment/ (41)
16 labour/ (3564)
17 forced labour/ (24)
18 part time labour/ (10)
19 unskilled labour/ (39)
20 attitudes to work/ (453)
21 work norms/ (39)
22 cleaning/ (2937)
23 child care/ (2219)
24 or/12-23 (52145)
25 exp housing/ (19665)
26 households/ (18224)
27 fostering/ (342)
28 foster family/ (28)
29 foster homes/ (53)
30 or/25-29 (37248)
31 child labour/ (321)
32 young workers/ (245)
33 migrant labour/ (980)
34 or/31-33 (1444)
35 housework/ (53)
36 child careproviders/ (548)
37 ((child* or boy? or girl? or schoolage? or schoolchild* or prepubescen* or pubescen* or puberty or adolescen* or juvenil* or underage* or teen or teens or teenage* or preteen* or pre-teen* or youth? or early adult* or emerging adult* or young adult* or young person or young people or minor? or offspring or sibling* or son or sons or daughter? or orphan* or migrant* or foreign* or transient) adj3 (labo?r* or physical* demand* or workday? or work or working or worker? or service? or exploit* or slavery or slave? or servitude or livelihood? or employ* or chores or cleaning or cooking or child-care or childcare)).ti,ab. (14780)
38 ((child* or boy? or girl? or schoolage? or schoolchild* or prepubescen* or pubescen* or puberty or adolescen* or juvenil* or underage* or teen or teens or teenage* or preteen* or pre-teen* or youth? or early adult* or emerging adult* or young adult* or young person or young people or minor? or offspring or sibling* or son or sons or daughter? or orphan* or migrant* or foreign* or transient) adj3 (domestic or household? or home or house or kin relations* or kinship or fostering or relocation or non-parental residence)).ti,ab. (7582)

39 ((labo?r* or physical* demand* or workday? or work or working or worker? or service? or exploit* or slavery or slave? or servitude or livelihood? or employ* or chores or cleaning or cooking or child-care or childcare) adj3 (domestic or household? or home or house or kin relations* or kinship or fostering or relocation or non-parental residence)).ti,ab. (6713)
40 (child* or boy? or girl? or schoolage? or schoolchild* or prepubescen* or pubescen* or puberty or adolescen* or juvenil* or underage* or teen or teens or teenage* or preteen* or pre-teen* or youth? or early adult* or emerging adult* or young adult* or young person or young people or minor? or offspring or sibling* or son or sons or daughter? or orphan* or migrant* or foreign* or transient).ti,ab. (514402)
41 (labo?r* or physical* demand* or workday? or work or working or worker? or service? or exploit* or slavery or slave? or servitude or livelihood? or employ* or chores or cleaning or cooking or child-care or child-care).ti,ab. (627133)
42 (domestic or household? or home or house or kin relations* or kinship or fostering or relocation or non-parental residence).ti,ab. (166198)
43 (housework or maid? or housemaid? or house help or domestic help).ti,ab. (690)
44 11 and 24 and 30 (905)
45 30 and 34 (72)
46 11 and 35 (13)
47 30 and 36 (23)
48 30 and 37 (835)
49 24 and 38 (410)
50 11 and 39 (1511)
51 35 and 40 (24)
52 34 and 43 (6)
53 ((child* or boy? or girl? or schoolage? or schoolchild* or prepubescen* or pubescen* or puberty or adolescen* or juvenil* or underage* or teen or teens or teenage* or preteen* or pre-teen* or youth? or early adult* or emerging adult* or young adult* or young person or young people or minor? or offspring or sibling* or son or sons or daughter? or orphan* or migrant* or foreign* or transient) adj3 (labo?r* or physical* demand* or workday? or work or working or worker? or service? or exploit* or slavery or slave? or servitude or livelihood? or employ* or chores or cleaning or cooking or child-care or childcare) adj3 (domestic or household? or home or house or kin relations* or kinship or fostering or relocation or non-parental residence)).ti,ab. (385)
54 ((child* or boy? or girl? or schoolage? or schoolchild* or prepubescen* or pubescen* or puberty or adolescen* or juvenil* or underage* or teen or teens or teenage* or preteen* or pre-teen* or youth? or early adult* or emerging adult* or young adult* or young person or young people or minor? or offspring or sibling* or son or sons or daughter? or orphan* or migrant* or foreign* or transient) adj3 (housework or maid? or housemaid? or house help or domestic help)).ti,ab. (37)
55 or/44-54 (3225)
56 exp australia/ (55032)
57 australia.ti,ab. (34512)
58 exp french polynesia/ (888)
59 guam/ (408)
60 (guam or northern mariana islands).ti,ab. (498)
61 new caledonia/ (667)
62 new caledonia.ti,ab. (822)
63 exp new zealand/ (15310)
64 new zealand.ti,ab. (14666)
65 exp japan/ (51803)
66 japan.ti,ab. (35694)
67 korea republic/ (23667)
68 ((korea or south korea or (republic adj2 korea)) not (north korea or (democratic adj2 korea))).ti,ab. (16454)

69 palau/ (106)
70 (palau or paulau).ti,ab. (135)
71 andorra/ (17)
72 andorra.ti,ab. (19)
73 cyprus/ (1192)
74 cyprus.ti,ab. (1198)
75 liechtenstein/ (44)
76 liechtenstein.ti,ab. (64)
77 monaco/ (48)
78 monaco.ti,ab. (62)
79 san marino/ (37)
80 san marino.ti,ab. (43)
81 austria/ (7825)
82 austria.ti,ab. (4830)
83 belgium/ (9772)
84 belgium.ti,ab. (6612)
85 czech republic/ (5650)
86 czech republic.ti,ab. (3445)
87 exp nordic countries/ (62545)
88 (denmark or faeroe islands).ti,ab. (9895)
89 sweden.ti,ab. (14928)
90 norway.ti,ab. (8941)
91 finland.ti,ab. (9004)
92 iceland.ti,ab. (1460)
93 scandinavia.ti,ab. (782)
94 (nordic country or nordic countries).ti,ab. (704)
95 exp baltic states/ (4245)
96 baltic states.ti,ab. (95)
97 estonia.ti,ab. (1316)
98 latvia.ti,ab. (981)
99 lithuania.ti,ab. (1389)
100 exp france/ (45673)
101 france.ti,ab. (30992)
102 exp germany/ (48193)
103 germany.ti,ab. (27540)
104 gibraltar/ (62)
105 gibraltar.ti,ab. (72)
106 exp greece/ (11682)
107 greece.ti,ab. (7587)
108 greenland/ (768)
109 greenland.ti,ab. (863)
110 hungary/ (7031)
111 hungary.ti,ab. (5005)
112 exp british isles/ (97493)
113 (ireland or eire).ti,ab. (5713)
114 (great britain or GBR or united kingdom or UK or northern ireland or scotland or channel islands or isle of man or (wales not new south wales) or (england not new england)).ti,ab. (66876)
115 exp italy/ (49717)
116 italy.ti,ab. (29711)

117 luxembourg/ (426)
118 luxembourg.ti,ab. (433)
119 netherlands/ (27631)
120 (netherlands or holland).ti,ab. (18361)
121 poland/ (21895)
122 poland.ti,ab. (13609)
123 exp portugal/ (7231)
124 portugal.ti,ab. (4990)
125 slovakia/ (3242)
126 (slovakia or slovak republic).ti,ab. (2812)
127 slovenia/ (3233)
128 slovenia.ti,ab. (2153)
129 exp spain/ (38436)
130 (spain or balearic islands or canary islands).ti,ab. (24355)
131 switzerland/ (13965)
132 switzerland.ti,ab. (8329)
133 exp "antigua and barbuda"/ (157)
134 (Antigua or Barbuda).ti,ab. (191)
135 argentina/ (12671)
136 argentina.ti,ab. (9602)
137 aruba/ (59)
138 aruba.ti,ab. (76)
139 bahamas/ (228)
140 bahamas.ti,ab. (242)
141 barbados/ (545)
142 barbados.ti,ab. (589)
143 british virgin islands/ (37)
144 british virgin islands.ti,ab. (31)
145 cayman islands/ (79)
146 cayman islands.ti,ab. (42)
147 chile/ (7580)
148 chile.ti,ab. (6347)
149 curacao/ (208)
150 curacao.ti,ab. (261)
151 puerto rico/ (3908)
152 puerto rico.ti,ab. (3020)
153 "saint martin (island)"/ (65)
154 (st martin or sint maarten).ti,ab. (50)
155 exp "trinidad and tobago"/ (2168)
156 (trinidad adj2 tobago).ti,ab. (598)
157 "turks and caicos islands"/ (25)
158 (turks adj2 caicos islands).ti,ab. (20)
159 exp virgin islands/ (303)
160 virgin islands.ti,ab. (324)
161 uruguay/ (2463)
162 uruguay.ti,ab. (2031)
163 exp "saint kitts and nevis"/ (146)
164 ((st kitts or saint kitts) adj2 nevis).ti,ab. (57)
165 exp panama/ (3155)

166 panama.ti,ab. (3635)
167 exp seychelles/ (327)
168 seychelles.ti,ab. (351)
169 bahrain/ (541)
170 bahrain.ti,ab. (454)
171 israel/ (10767)
172 israel.ti,ab. (7014)
173 kuwait/ (1871)
174 kuwait.ti,ab. (1556)
175 malta/ (1026)
176 malta.ti,ab. (1028)
177 oman/ (1248)
178 oman.ti,ab. (1045)
179 qatar/ (744)
180 (qatar or quatar or katar).ti,ab. (608)
181 saudi arabia/ (9435)
182 saudi arabia.ti,ab. (7316)
183 exp united arab emirates/ (1327)
184 (united arab emirates or trucial states or abu dhabi or ajman or dubai or fujairah or ras al-khaimah or sharjah or umm al-qaiwain).ti,ab. (1129)
185 bermuda/ (138)
186 bermuda.ti,ab. (534)
187 exp canada/ (47146)
188 canada.ti,ab. (27900)
189 exp usa/ (303207)
190 (united states or (united states adj2 america) or USA).ti,ab. (127438)
191 or/56-190 (991990)
192 55 not 191 (1875)

3.5 OvidSP EconLit

Database name	EconLit
Database platform	OvidSP
Dates of database coverage	1886 to May 23, 2019
Date searched	05/06/2019
Searched by	JF
Number of hits	483

1 ((child* or boy? or girl? or schoolage? or schoolchild* or prepubescen* or pubescen* or puberty or adolescen* or juvenil* or underage* or teen or teens or teenage* or preteen* or pre-teen* or youth? or early adult* or emerging adult* or young adult* or young person or young people or minor? or offspring or sibling* or son or sons or daughter? or orphan* or migrant* or foreign* or transient) adj3 (labo?r* or physical* demand* or work-

day? or work or working or worker? or service? or exploit* or slavery or slave? or servitude or livelihood? or employ* or chores or cleaning or cooking or child-care or childcare) adj3 (domestic or household? or home or house or kin relations* or kinship or fostering or relocation or non-parental residence)).ti,ab. (545)

2 ((child* or boy? or girl? or schoolage? or schoolchild* or prepubescen* or pubescen* or puberty or adolescen* or juvenil* or underage* or teen or teens or teenage* or preteen* or pre-teen* or youth? or early adult* or emerging adult* or young adult* or young person or young people or minor? or offspring or sibling* or son or sons or daughter? or orphan* or migrant* or foreign* or transient) adj3 (housework or maid? or housemaid? or house help or domestic help)).ti,ab. (76)

3 1 or 2 (609)

4 (australia or french polynesia or guam or new caledonia or new zealand).ti,ab. (14148)

5 (japan or ((korea or south korea or (republic adj2 korea)) not (north korea or (democratic adj2 korea))) or palau or paulau).ti,ab. (25239)

6 (andorra or cyprus or liechtenstein or monaco or san marino or austria or belgium or czech republic or nordic countries or nordic country or denmark or faeroe islands or sweden or norway or finland or iceland or scandinavia or baltic states or estonia or latvia or lithuania or france or germany or gibraltar or greece or greenland or hungary or great britain or gbr or united kingdom or uk or northern ireland or scotland or channel islands or isle of man or (wales not new south wales) or (england not new england) or italy or luxembourg or netherlands or holland or poland or portugal or slovakia or slovak republic or slovenia or spain or balearic islands or canary islands or switzerland).ti,ab. (112253)

7 (antigua or baruba or argentina or aruba or bahamas or barbados or virgin islands or cayman islands or chile or curacao or puerto rico or st martin or sint maarten or (trinidad adj2 tobago) or (turks adj2 caicos islands) or uruguay or ((st kitts or saint kitts) adj2 nevis) or panama).ti,ab. (11446)

8 seychelles.ti,ab. (45)

9 (bahrain or israel or kuwait or malta or oman or qatar or quatar or katar or saudi arabia or united arab emirates or trucial states or abu dhabi or ajman or dubai or fujairah or ras al-khaimah or sharjah or umm al-qaiwain or bermuda).ti,ab. (4828)

10 (canada or united states or usa).ti,ab. (59749)

11 or/4-10 (205623)

12 3 not 11 (483)

3.6 Web of Science Core Collection

Database name	Web of Science Core Collection
Database platform	Clarivate Analytics
Dates of database coverage	Science Citation Index Expanded (SCI-EXPANDED) --1970-present<br>Social Sciences Citation Index (SSCI) --1970-present<br>Arts & Humanities Citation Index (A&HCI) --1975-present<br>Conference Proceedings Citation Index- Science (CPCI-S) --1990-present<br>Conference Proceedings Citation Index- Social Science & Humanities (CPCI-SSH) --1990-present<br>Emerging Sources Citation Index (ESCI) --2015-present<br><br>Data last updated: 2019/06/04

Date searched	05/06/2019
Searched by	JF
Number of hits	2224

1 TOPIC: ((child* or boy or girl or schoolage$ or schoolchild* or prepubescen* or pubescen* or puberty or adolescen* or juvenil* or underage* or "teen" or "teens" or teenage* or preteen* or pre-teen* or youth$ or "early adult*" or "emerging adult*" or "young adult*" or "young person" or "young people" or minor$ or offspring or sibling* or "son" or "sons" or daughter$ or orphan* or migrant* or foreign* or transient) NEAR/3 ("labo$r" or "physical* demand*" or workday$ or "work" or "working" or worker? or service$ or exploit* or "slavery" or slave? or "servitude" or "servant" or livelihood$ or employ* or "chores" or "cleaning" or "cooking" or "child-care" or "childcare") NEAR/3 ("domestic" or "house" or "home" or household$ or "kin relations*" or "kinship" or "fostering" or "relocation" or "non-parental residence")) (2,725)

2 TOPIC: ((child* or boy or girl or schoolage$ or schoolchild* or prepubescen* or pubescen* or puberty or adolescen* or juvenil* or underage* or "teen" or "teens" or teenage* or preteen* or pre-teen* or youth$ or "early adult*" or "emerging adult*" or "young adult*" or "young person" or "young people" or minor$ or offspring or sibling* or "son" or "sons" or daughter$ or orphan* or migrant* or foreign* or transient) NEAR/3 (housework* or maid$ or housemaid$ or "house help" or "domestic help")) (369)

3 #1 OR #2 (3,041)

4 TOPIC: ("australia" or "french polynesia" or "guam" or "new caledonia" or "new zealand") (349,898)

5 TOPIC: ("japan" or (("korea" or "south korea" or ("republic" NEAR/2 "korea")) not ("north korea" or ("democratic" NEAR/2 "korea"))) or "palau" or "paulau") (357,669)

6 TOPIC: ("andorra" or "cyprus" or "liechtenstein" or "monaco" or "san marino" or "austria" or "belgium" or "czech republic" or "nordic countries" or "nordic country" or "denmark" or "faeroe islands" or "sweden" or "norway" or "finland" or "iceland" or "scandinavia" or "baltic states" or "estonia" or "latvia" or "lithuania" or "france" or "germany" or "gibraltar" or "greece" or "greenland" or "hungary" or "great britain" or "gbr" or "united kingdom" or "uk" or "northern ireland" or "scotland" or "channel islands" or "isle of man" or ("wales" not "new south wales") or ("england" not "new england") or "italy" or "luxembourg" or "netherlands" or "holland" or "poland" or "portugal" or "slovakia" or "slovak republic" or "slovenia" or "spain" or "balearic islands" or "canary islands" or "switzerland") (1,694,134)

7 TOPIC: ("antigua" or "baruba" or "argentina" or "aruba" or "bahamas" or "barbados" or "virgin islands" or "cayman islands" or "chile" or "curacao" or "puerto rico" or "st martin" or "sint maarten" or ("trinidad" NEAR/2 "tobago") or ("turks" NEAR/2 "caicos islands") or "uruguay" or (("st kitts" or "saint kitts") NEAR/2 "nevis") or "panama") (147,347)

8 TOPIC: ("seychelles") (2,366)

9 TOPIC: ("bahrain" or "israel" or "kuwait" or "malta" or "oman" or "qatar" or "quatar" or "katar" or "saudi arabia" or "united arab emirates" or "trucial states" or "abu dhabi" or "ajman" or "dubai" or "fujairah" or "ras al-khaimah" or "sharjah" or "umm al-qaiwain" or "bermuda") (119,062)

10 TOPIC: ("canada" or "united states" or "usa") (959,637)

11 #4 OR #5 OR #6 OR #7 OR #8 OR #9 OR #10 (3,420,302)

12 #3 NOT #11 (2,224)

3.7 IBSS

Database name	IBSS (International Bibliography of the Social Sciences)
Database platform	ProQuest

Dates of database coverage	Complete database to 05/06/2019
Date searched	05/06/2019
Searched by	JF
Number of hits	1,400

Two searches were carried out and results from both were uploaded to EndNote as the system was unable to combine the two searches with the Boolean operator OR.

TI,AB((child* OR boy OR girl OR schoolage$ OR schoolchild* OR prepubescen* OR pubescen* OR puberty OR adolescen* OR juvenil* OR underage* OR "teen" OR "teens" OR teenage* OR preteen* OR pre-teen* OR youth$ OR "early adult*" OR "emerging adult*" OR "young adult*" OR "young person" OR "young people" OR minor$ OR offspring OR sibling* OR "son" OR "sons" OR daughter$ OR orphan* OR migrant* OR foreign* OR transient) NEAR/3 ("labo$r" OR "physical* demand*" OR workday$ OR "work" OR "working" OR worker? OR service$ or exploit* OR "slavery" OR slave? OR "servitude" OR "servant" OR livelihood$ OR employ* OR "chores" OR "cleaning" OR "cooking" OR "childcare" OR "childcare") NEAR/3 ("domestic" OR "house" OR "home" OR household$ OR "kin relations*" OR "kinship" OR "fostering" OR "relocation" OR "non-parental residence")) NOT TI,AB("australia" OR "french polynesia" OR "guam" OR "new caledonia" OR "new zealand" OR "japan" OR (("korea" OR "south korea" OR ("republic" NEAR/2 "korea")) NOT ("north korea" OR ("democratic" NEAR/2 "korea"))) OR "palau" OR "paulau" OR "andorra" OR "cyprus" OR "liechtenstein" OR "monaco" OR "san marino" OR "austria" OR "belgium" OR "czech republic" OR "nordic countries" OR "nordic country" OR "denmark" OR "faeroe islands" OR "sweden" OR "norway" OR "finland" OR "iceland" OR "scandinavia" OR "baltic states" OR "estonia" OR "latvia" OR "lithuania" OR "france" OR "germany" OR "gibraltar" OR "greece" OR "greenland" OR "hungary" OR "great britain" OR "gbr" OR "united kingdom" OR "uk" OR "northern ireland" OR "scotland" OR "channel islands" OR "isle of man" OR ("wales" NOT "new south wales") OR ("england" NOT "new england") OR "italy" OR "luxembourg" OR "netherlands" OR "holland" OR "poland" OR "portugal" OR "slovakia" OR "slovak republic" OR "slovenia" OR "spain" OR "balearic islands" OR "canary islands" OR "switzerland" OR "antigua" OR "baruba" OR "argentina" OR "aruba" OR "bahamas" OR "barbados" OR "virgin islands" OR "cayman islands" OR "chile" OR "curacao" OR "puerto rico" OR "st martin" OR "sint maarten" OR ("trinidad" NEAR/2 "tobago") OR ("turks" NEAR/2 "caicos islands") OR "uruguay" OR (("st kitts" OR "saint kitts") NEAR/2 "nevis") OR "panama" OR "seychelles" OR "bahrain" OR "israel" OR "kuwait" OR "malta" OR "oman" OR "qatar" OR "quatar" OR "katar" OR "saudi arabia" OR "united arab emirates" OR "trucial states" OR "abu dhabi" OR "ajman" OR "dubai" OR "fujairah" OR "ras al-khaimah" OR "sharjah" OR "umm al-qaiwain" OR "bermuda" OR "canada" OR "united states" OR "usa") (1176)

TI,AB((child* OR boy OR girl OR schoolage$ OR schoolchild* OR prepubescen* OR pubescen* OR puberty OR adolescen* OR juvenil* OR underage* OR "teen" OR "teens" OR teenage* OR preteen* OR pre-teen* OR youth$ OR "early adult*" OR "emerging adult*" OR "young adult*" OR "young person" OR "young people" OR minor$ OR offspring OR sibling* OR "son" OR "sons" OR daughter$ OR orphan* OR migrant* OR foreign* OR transient) NEAR/3 (housework* OR maid$ OR housemaid$ OR "house help" OR "domestic help")) NOT TI,AB("australia" OR "french polynesia" OR "guam" OR "new caledonia" OR "new zealand" OR "japan" OR (("korea" OR "south korea" OR ("republic" NEAR/2 "korea")) NOT ("north korea" OR ("democratic" NEAR/2 "korea"))) OR "palau" OR "paulau" OR "andorra" OR "cyprus" OR "liechtenstein" OR "monaco" OR "san marino" OR "austria" OR "belgium" OR "czech republic" OR "nordic countries" OR "nordic country" OR "denmark" OR "faeroe islands" OR "sweden" OR "norway" OR "finland" OR "iceland" OR "scandinavia" OR "baltic states" OR "estonia" OR "latvia" OR "lithuania" OR "france" OR "germany" OR "gibraltar" OR "greece" OR "greenland" OR "hungary" OR "great britain" OR "gbr" OR "united kingdom" OR "uk" OR "northern ireland" OR "scotland" OR "channel islands" OR "isle of man" OR ("wales" NOT "new south wales") OR ("england"

NOT "new england") OR "italy" OR "luxembourg" OR "netherlands" OR "holland" OR "poland" OR "portugal" OR "slovakia" OR "slovak republic" OR "slovenia" OR "spain" OR "balearic islands" OR "canary islands" OR "switzerland" OR "antigua" OR "baruba" OR "argentina" OR "aruba" OR "bahamas" OR "barbados" OR "virgin islands" OR "cayman islands" OR "chile" OR "curacao" OR "puerto rico" OR "st martin" OR "sint maarten" OR ("trinidad" NEAR/2 "tobago") OR ("turks" NEAR/2 "caicos islands") OR "uruguay" OR (("st kitts" OR "saint kitts") NEAR/2 "nevis") OR "panama" OR "seychelles" OR "bahrain" OR "israel" OR "kuwait" OR "malta" OR "oman" OR "qatar" OR "quatar" OR "katar" OR "saudi arabia" OR "united arab emirates" OR "trucial states" OR "abu dhabi" OR "ajman" OR "dubai" OR "fujairah" OR "ras al-khaimah" OR "sharjah" OR "umm al-qaiwain" OR "bermuda" OR "canada" OR "united states" OR "usa")
(224)

Jane Falconer
Information Support Services Librarian
07/06/2019

3.8 ILO Labourdoc (grey)

Database searched using date limits 1990-2019

Database name	ILO Labourdoc
Database platform	Online website
Dates of database coverage	Complete database to 28/06/2019
Date searched	28/06/2019
Searched by	NP
Number of hits	96 (English: 78)

#1 Any field is (exact) “child domestic work”

#2 Filter: Years 1990 – 2019 (96)

#3 Filter: English language (78)

3.9 Understanding Children’s Work

http://www.ucw-project.org/research.aspx?search=child%20domestic%20work

Database name	Understanding Children’s Work
Database platform	Online website

Dates of database coverage	?
Keyword	"Child Domestic Worker", no filters applied
Date searched	03/07/2019
Searched by	CC
Number of hits	6

3.10 Freedom Fund library

https://resourcecentre.savethechildren.net

Database name	Save the Children
Database platform	Online website
Dates of database coverage	?
Keyword	"Child Domestic Worker", no filters applied
Date searched	03/07/2019
Searched by	CC
Number of hits	6

3.11 Anti-Slavery International CDW reports:

https://www.antislavery.org/reports-and-resources/research-reports/domestic-work-slavery-reports/

Database name	Understanding Children's Work
Database platform	Online website
Dates of database coverage	?
Keyword	"Child Domestic Worker", no filters applied
Date searched	03/07/2019
Searched by	CC

Number of hits	11

3.12 Young lives publications:

https://www.younglives.org.uk/content/publications-0

#1 Keyword: "child domestic worker"

Total: 434 results (please note: numbers don't match up as some articles may be tagged under more than one theme)

Initial Breakdown of Results

Theme:

- Poverty and inequality (149)
- Nutrition, health and well-being (135)
- Education (85)
- Methods (72)
- Child protection (55)
- Gender, adolescence & youth (48)
- Skills & Work (22)

Type:

- Working paper (123)
- Journal Article (119)
- Policy paper (57)
- Technical notes (30)
- Book / chapter (28)
- Country report (28)
- Student paper (26)
- Summative Output (13)
- Impact case study (8)
- Resources for teachers (1)
- Story (1)

#2 Filter by theme: "Skills & Work" – 22 results

1. Working paper – 11 (1 duplicate)
2. Journal article – 3 (0 duplicates)
3. Student paper – 2 (0 duplicates)
4. Book/chapters – 3 (0 duplicates)
5. Summative output – 2 (0 duplicates)
